# Supplementary material for: Identification of Freezing-Responsive microRNAs and Their Targets in Chinese Jujube by Small RNA and Degradome Sequencing
Source: Int J Mol Sci. 2026 Sep 4;27(17):7912. doi: 10.3390/ijms27177912 (PMC13566890; doi:10.3390/ijms27177912)
Supplement: Supplementary file 1 [file ijms-27-07912-s001.zip › Supplementary Material S2/novel_miR_77.pdf]

Provisional ID	:	novel_miR_77
Score total	:	7166.8
Score for star read(s)	:	3.9
Score for read counts	:	7158.1
Score for mfe	:	3.3
Score for randfold	:	1.6
Score for cons. seed	:	
Total read count	:	14052
Mature read count	:	9107
Loop read count	:	29
Star read count	:	4916

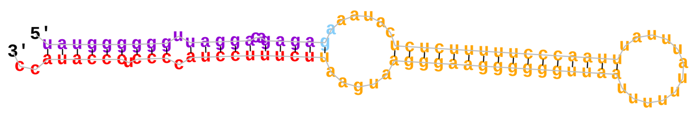

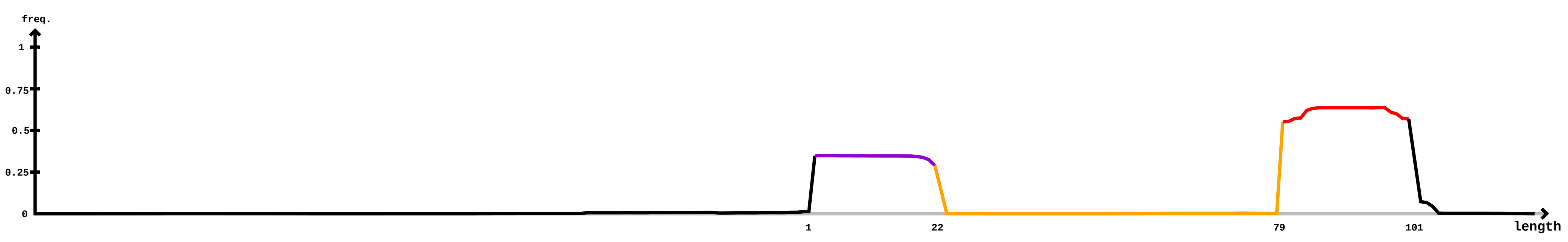

Star Mature

```
5'- ccuuuuuuucaccaaaaucuagacucaauuaauuucucuuuucccuuccuccaccaucugguguauuucuuuucucacuaaaaggauggaagagggaaaggcuuaagagugugggguggaagugauagguaugggggguuaggacagagagaaauacucucuuuuucccaauuuauuuauuuuuuaauuggggggaagggaaugaauucuuuccuaccccucccauaccauucauuuccauccuuuccuuc -3'  obs
    ccuuuuuuucaccaaaaucuagacucaauuaauuucucuuuucccuuccuccaccaucugguguauuucuuuucucacuaaaaggauggaagagggaaaggcuuaagagugugggguggaagugauagguaugggggguuaggacagagagaaauacucucuuuuucccaauuuauuuauuuuuuaauuggggggaagggaaugaauucuuuccuaccccucccauaccauucauuuccauccuuuccuuc      exp
    ...................................((((((((((..((((((...(((((...........)))))...)).)))).))))))))))...(((.(...((((((((((((..(((((((((((.(((((..(((((......((((((((((((((((...........))))))))))))))))).....))))))))))).))).))))))))..))))))))))))...)))).
```

read	reads	mm	sample
caccaaaaucuagacucaauu	1	0	LT-1
ccaaaaucuagacucaauuaauuucucu	1	0	JCK-2
aaucuagacucaauuaauuucuc	1	0	LT-3
uagacucaauuaauuucucuuuuc	1	0	JCK-1
agacucaauuaauuucucuuuucc	1	0	JCK-2
agacucaauuaauuucucuuuucc	1	0	JCK-3
agacucaauuaauuucucuuuucc	1	0	JCK-1
ucugguguauuucuuuucuc	2	0	JCK-3
ucucacuaaaaggauggaagagggaa	2	0	LT-1
ucucacuaaaaggauggaagagggaaagg	1	0	JCK-1
ucucacuaaaaggauggaagagggaaagg	1	0	JCK-3
cucacuaaaaggauggaagagggaaagg	1	0	JCK-3
cacuaaaaggauggaagagg	1	0	LT-3
cacuaaaaggauggaagagggaaagg	1	0	JCK-2
cacuaaaaggauggaagagggaaaggA	2	1	JCK-2
cacuaaaaggauggaagagggaaaggA	2	1	JCK-3
aaaaggauggaagagggaaagg	1	0	LT-3
aaaaggauggaagagggaaagg	4	0	LCK-3
aaaggauggaagagggaaagg	1	0	LT-3
aggauggaagagggaaagg	3	0	LT-2
agagggaaaggcuuaagagu	5	0	LT-2
agagggaaaggcuuaagagug	1	0	LT-3
agagggaaaggcuuaagagugu	36	0	LCK-2
agagggaaaggcuuaagagugu	21	0	LCK-1
gagggaaaggcuuaagagug	1	0	JCK-2
Cgggaaaggcuuaagagugugg	1	1	LT-1
aaaggcuuaagagugugggugg	1	0	LT-3
aaaggcuuaagagugugggguggaagu	2	0	JCK-1
aggcuuaagagugugggug	1	0	LT-1
cuuaagagugugggguggaaguga	2	0	JT-2
cuuaagagugugggguggaaguga	2	0	JT-3
cuuaagagugugggguggaagugauag	2	0	JCK-3
cuuaagagugugggguggaagugauag	2	0	JCK-2

Star (purple) — **Mature** (red)

Precursor: ccuuuuuuucaccaaaaucuagacucaauuaauuucucuuuucccuuccuccaccaucugguguauuucuuuucucacuaaaaggauggaagagggaaaggcuuaagaguguggguggaagugauagg**uaugggggguuaggacagaga**gaaauacucucuuuucccaauuuauuuauuuuuuaauuggggggaagggaaugaau**ucuuuccuaccccucccauacc**auucauuuccauccuuuccuuc

Read	Count	Mismatch	Library
cuuaagaguguggguggaagugauagg	5	0	JCK-2
cuuaagaguguggguggaagugauagg	1	0	LT-2
cuuaagaguguggguggaagugauagg	3	0	JT-3
cuuaagaguguggguggaagugauagg	1	0	JCK-3
cuuaagaguguggguggaagugauagg	4	0	JT-2
cuuaagaguguggguggaagugauagg	2	0	LT-1
aagaguguggguggaagugauagg	4	0	JT-3
aagaguguggguggaagugauagg	2	0	JT-2
guguggguggaagugauagg	1	0	JCK-1
guguggguggaagugauagg	1	0	JCK-3
uguggguggaagugauaggu	10	0	LCK-3
guggguggaagugauagguau	1	0	LT-1
guggguggaagugauagguaug	8	0	LCK-3
uggguggaagugauagguaugg	2	0	JCK-3
gguggaagugauagguauggg	1	0	LT-3
guggaagugauagguaug	5	0	LCK-3
guggaagugauagguauggg	1	0	LT-1
guggaagugauagguaugggg	2	0	JCK-2
guggaagugauagguaugggg	1	0	JCK-3
uggaagugauagguaugg	1	0	LT-3
uggaagugauagguaugggg	2	0	JCK-2
uggaagugauagguaugggg	1	0	JCK-3
uggaagugauagguaugggggg	6	0	LCK-3
agugauagguaugggggguu	3	0	LT-3
agugauagguaugggggguuaggaca	7	0	LCK-3
agugauagguaugggggguuaggacagaga	2	0	LCK-3
ugauagguaugggggguuaggaca	3	0	LT-3
auagguaugggggguuaggacaga	1	0	JCK-2
auagguaugggggguuaggacaga	1	0	JCK-1
uagguaugggggguuagga	4	0	LT-3
uagguaugggggguuagga	1	0	JCK-1
uagguaugggggguuaggac	2	0	JCK-2
uagguaugggggguuaggacU	1	1	LT-3
uagguaugggggguuaggaca	4	0	LCK-3
uagguaugggggguuaggacC	1	1	LT-3
uagguaugggggguuaggaca	1	0	LT-3
uagguaugggggguuaggaca	3	0	JT-3
uagguaugggggguuaggaca	3	0	LT-1
uagguaugggggguuaggaca	6	0	JCK-2
uagguaugggggAuuaggaca	1	1	LT-1
uagguaugggggguuaggaca	1	0	JT-2
uagguaugggggguuaggaca	1	0	JCK-1
uagguaugggggguuaggaca	6	0	JCK-3
uagguaugggggguuaggacC	2	1	JCK-3
uagguaugggggguuaggacaga	1	0	JCK-3
gguaugggggguuaggacag	1	0	JCK-3
gguaugggggguuaggacaga	4	0	LT-1
gguaugggggguuaggacaga	9	0	JCK-1
gguaugggggguuaggacaga	3	0	LT-3
gguaugggggguuaggacaga	4	0	JCK-3
gguaugggggguuaggacaga	1	0	LCK-3
gguaugggggguuaggacaga	12	0	JT-1
gguaugggggguuaggacaga	9	0	LT-2
gguaugggggguuaggacaga	3	0	JCK-2
guaugggggguuaggacaga	6	0	LCK-3
guaugggggguuaggacaga	1	0	LT-3
guaugggggguuaggacaga	2	0	LT-1
guaugggggguuaggacagag	1	0	JCK-3
Cuaugggggguuaggacagaga	2	1	JCK-2
Cuaugggggguuaggacagaga	1	1	LT-3
Cuaugggggguuaggacagaga	7	1	JT-1
Cuaugggggguuaggacagaga	2	1	JCK-1
uaugggggguuaggacag	10	0	LT-3
uaugggggguuaggacag	8	0	LT-1
uaugggggguuaggacag	9	0	JCK-1
uaugggggguuaggacag	10	0	LCK-3
uaugggggguuaggacag	14	0	JCK-2
uaugggggguuaggacag	18	0	JCK-3
uaugggggguuCggacag	1	1	JCK-2
uaugggggguuaggacag	8	0	LT-2

	Star		Mature	
ccuuuuuuucaccaaaaucuagacucaauuaauuucucuuuucccuuccuccaccaucugguguauuucuuuucucacuaaaaggauggaagagggaaaggcuuaagaguguggguggaagugauagg	uaugggggguuaggacagaga	gaaauacucucuuuucccaauuuauuuauuuuuuaauuggggggaagggaaugaau	ucuuuccuaccccucccauacc	auucauuuccauccuuuccuuc

Read			
...........uaugggggguuaggacaga...........	11	0	JT-2
...........uaugggggguuaggacaga...........	19	0	LT-2
...........uaugggggguuaggacaga...........	11	0	JT-1
...........uaugggggguuaggacaga...........	11	0	JCK-2
...........uaugggggguuaggacaga...........	5	0	JT-3
...........uNugggggguuaggacaga...........	1	1	JT-2
...........uaugggggguuaggacaga...........	12	0	JCK-3
...........uaugggggguuaggacagU...........	1	1	LT-1
...........uaugggggguuaggacaga...........	8	0	LT-1
...........uaugggggguuaggacagU...........	1	1	JCK-2
...........uaugggggguuaggacaga...........	37	0	LCK-3
...........uCugggggguuaggacaga...........	1	1	JCK-1
...........uaugggggguuaggacaga...........	14	0	LT-3
...........uaugggggguuaggacagag...........	115	0	JT-1
...........uaugggggguuaggacagag...........	20	0	JT-3
...........uaugggggguuaggacagag...........	60	0	JCK-1
...........uCugggggguuaggacagag...........	1	1	JT-3
...........uCugggggguuaggacagag...........	3	1	JCK-2
...........uaugggggguuaggacagag...........	43	0	LT-3
...........uaugggggguuaggacagag...........	7	0	JT-2
...........uaugggggguuaggacagaN...........	1	1	JCK-1
...........uaugggggguuaggacagaU...........	1	1	LT-2
...........uaugggggguuCggacagag...........	1	1	JCK-2
...........uaugggggguuNggacagag...........	1	1	JCK-1
...........uaugggggguuaggacagag...........	16	0	LCK-3
...........uaugggggguuaggacagag...........	35	0	LT-2
...........uaugggggguuaggacagag...........	34	0	LT-1
...........uaugggggguuaggacagag...........	62	0	JCK-3
...........uaugggggguuaggacagag...........	91	0	JCK-2
...........uauAggggguuaggacagaga...........	1	1	JCK-1
...........uaugggUgguuaggacagaga...........	1	1	JT-3
...........uCugggggguuaggacagaga...........	4	1	LT-2
...........uaugggggguuagUacagaga...........	1	1	LCK-2
...........uaugggggguuagNacagaga...........	2	1	LCK-1
...........uaugggggguuaggacagaga...........	589	0	JT-1
...........uaugggggguuaggacagaga...........	555	0	JCK-3
...........uaugggggguuCggacagaga...........	1	1	LCK-2
...........uaugggggguuaggacagaga...........	727	0	JCK-2
...........uCugggggguuaggacagaga...........	1	1	JCK-3
...........uaugggggguuaggacagaga...........	88	0	LCK-2
...........uaugggggguuaggacagaga...........	289	0	LT-3
...........Caugggggguuaggacagaga...........	1	1	LT-2
...........uauAggggguuaggacagaga...........	1	1	JCK-2
...........uaugggggguuaggacagaga...........	192	0	JT-2
...........uaugggggguuaggacagagC...........	2	1	JCK-1
...........uCugggggguuaggacagaga...........	1	1	JT-3
...........uaugggggguuaggacagaga...........	242	0	LT-2
...........uaugggggguuaggCcagaga...........	1	1	JCK-1
...........uaugggggguuaggacagaga...........	345	0	LCK-3
...........uaugggggguuaggacagaga...........	478	0	JCK-1
...........uaugggggguuaggCcagaga...........	2	1	JCK-2
...........uaugggggguuaggCcagaga...........	1	1	LT-3
...........uaugggggguuaggacagaNa...........	1	1	JCK-1
...........uaCgggggguuaggacagaga...........	1	1	JT-3
...........uaugggggguuagAacagaga...........	1	1	LCK-3
...........uaugggggguuaggCcagaga...........	1	1	JCK-3
...........uaugggggguuNggacagaga...........	1	1	JCK-1
...........uCugggggguuaggacagaga...........	3	1	JT-1
...........uaugggggguuaggacagaga...........	113	0	LCK-1
...........uauggggggCuaggacagaga...........	1	1	JT-2
...........uaugggggguuaggacagagU...........	3	1	LT-3
...........uauAggggguuaggacagaga...........	1	1	JT-1
...........uaugggggguuaggacCgaga...........	1	1	JCK-2
...........uaugggggguuaggacagaga...........	206	0	LT-1
...........uNugggggguuaggacagaga...........	1	1	JCK-2
...........uaugggggguuaggacagCga...........	1	1	LT-2
...........uCugggggguuaggacagaga...........	2	1	LCK-3
...........uaugggggguuaggacagagC...........	2	1	JCK-3
...........uauAggggguuaggacagaga...........	1	1	JCK-3
...........uaugggggguuaggacagagU...........	1	1	JCK-1

Star | Mature

ccuuuuuuucaccaaaaucuagacucaauuaauuucucuuuucccuuccuccaccaucugguguauuucuuuucucacuaaaaggauggaagagggaaaggcuuaagagugugggguggaagugauagg**uaugggggguuaggacagaga**gaaauacucucuuuucccaauuuauuuauuuuuuaauuggggggaagggaaugaau**ucuuuccuaccccucccauacc**auucauuuccauccuuuccuuc

Read	Count	Mismatch	Library
uaugggggguuaggCcagaga	1	1	JT-2
uCugggggguuaggacagaga	6	1	JCK-2
uaNgggggguuaggacagaga	1	1	LCK-2
uaugggggguuaggCcagaga	1	1	JT-1
uaugggggguuaggacagaga	231	0	JT-3
uaugggggguuaggCcagaga	1	1	LCK-1
uaugggggguuCggacagaga	1	1	JCK-3
uaugggggguuCggacagaga	1	1	LCK-3
uaugggggguuaggacagagU	1	1	LT-1
uaugggggguuaggacagagaC	3	1	JCK-2
uaugggggguuaggacagagaU	1	1	JCK-2
uaugggggguuaggacagagaU	1	1	LT-2
uaugggggguuaggacagagaU	1	1	LT-3
uaugggggguuaggacagagaC	1	1	JCK-3
uaugggggguuaggacagagaU	2	1	LT-1
uaugggggguuaggacagagagaa	2	0	JCK-2
augggggguuaggacaga	1	0	LT-3
augggggguuaggacaga	2	0	LT-2
augggggguuaggacagag	3	0	JCK-1
augggggguuaggacagag	1	0	JCK-2
augggggguuaggacagag	1	0	LT-2
augggggguuaggacagaga	2	0	JCK-2
augggggguuaggacagaga	5	0	JCK-3
augggggguuaggacagagaU	1	1	JCK-2
ugggggguuaggacagaga	3	0	JCK-2
ugggggguuaggacagaga	1	0	JCK-1
gggggguuaggacagagagaaaua	1	0	LT-3
ggggguuaggacagagagaaauac	1	0	LT-3
gggguuaggacagagagaaauac	4	0	LCK-3
guuaggacagagagaaauacu	3	0	LCK-3
uuuuuaauuggggggaaggga	5	0	LCK-3
uuuuuaCuuggggggaaggga	1	1	LCK-3
uuaauuggggggaagggaau	1	0	LT-3
uuaauuggggggaCgggaaug	1	1	LT-1
uuaauuggggggaagggaaug	1	0	LT-1
uaauuggggggaagggaaugaau	1	0	LT-3
uaauuggggggaagggaaugaauA	6	1	LCK-3
uaauuggggggaagggaaugaauuc	2	0	LCK-3
uaauuggggggaagggaaugaauuc	2	0	LT-3
aauuggggggaagggaau	6	0	LCK-3
uuggggggaagggaaugaauucuuuc	7	0	LCK-3
ugggggaagggaaugaauucu	1	0	JCK-1
ugggggaagggaaugaauucu	1	0	JCK-2
ggggaagggaaugaauucu	1	0	LT-1
aaugaauucuuuccuaccccuc	4	0	JT-2
aaugaauucuuuccuaccccuc	7	0	JT-3
Cucuuuccuaccccuccca	1	1	LT-1
ucuuuccuacAccuccca	1	1	LCK-3
ucuuuccuaccccuccca	50	0	LT-3
ucuuuccuaccccuccca	9	0	JT-3
ucuuuccuaccccuccca	16	0	JCK-1
ucuuuccuaccccuccca	35	0	LCK-2
ucuuuccuaccccNccca	1	1	LCK-1
ucuuuccuaccccuccca	3	0	JT-1
ucuuuccuaccccuccca	88	0	LT-2
ucuuuccuaccccuccca	40	0	LT-1
ucuuuccuaccccuccca	9	0	JT-2
ucuuuccuaccccuccca	9	0	JCK-3
ucuuuccuaccccucccU	1	1	LCK-3
ucuuuccuaccccuccca	22	0	JCK-2
ucuuuccuaccccuccca	67	0	LCK-3
ucuuCccuaccccuccca	1	1	LT-2
ucuuuccuUccccuccca	3	1	LT-2
ucuuuccuaccccuccca	33	0	LCK-1
ucuuuccuaccccucccau	17	0	LT-1
ucuuuccuaccccucccau	25	0	LT-3
ucuuuccuaccccucccau	14	0	LT-2
ucuuuccuaccccuGccau	1	1	LCK-1
ucuuuccuaccccucccau	6	0	JCK-3
ucuuuccuaccccucccau	14	0	JCK-2

```
                                                                                                                                                                                                                Star                                                           Mature
ccuuuuuuucaccaaaaucuagacucaauuaauuucucuuuucccuuccuccaccaucugguguauuucuuuucucacuaaaaggauggaagagggaaaggcuuaagagugugggugguaagugauagguaugggggguuaggacagagagaaauacucucuuuuucccaauuuauuuauuuuuuaauuggggggaagggaaugaauucuuuccuaccccucccauaccauucauuuccauccuuuccuuc
..............................................................................................................................................................................................................ucuuuccuaccccucccau.........................   27     0     LCK-1
..............................................................................................................................................................................................................ucuuuccuaccccucccau.........................   26     0     LCK-2
..............................................................................................................................................................................................................ucuuuccuaccccucccau.........................   12     0     JCK-1
..............................................................................................................................................................................................................ucuuuccuaccccucccau.........................   23     0     LCK-3
..............................................................................................................................................................................................................ucuuuccuaccccuAccau.........................   1      1     LCK-3
..............................................................................................................................................................................................................ucuuuccuacUccucccaua........................   28     1     JT-1
..............................................................................................................................................................................................................ucuuuccuaccccucccCua........................   1      1     LT-3
..............................................................................................................................................................................................................ucuuuccuaccccucccaua........................   43     0     JCK-1
..............................................................................................................................................................................................................uNuuuccuaccccucccaua........................   1      1     JCK-2
..............................................................................................................................................................................................................ucuuuccuaccccucccauU........................   2      1     LT-3
..............................................................................................................................................................................................................ucuuuccuaccccucccaua........................   53     0     LCK-3
..............................................................................................................................................................................................................ucuuuccuaccccucccaua........................   59     0     JCK-2
..............................................................................................................................................................................................................ucuuuccuaccccucccaua........................   57     0     JCK-3
..............................................................................................................................................................................................................ucuuuccuaccccucccaAa........................   1      1     JCK-3
..............................................................................................................................................................................................................ucuuuccuaccccucccaua........................   35     0     LT-3
..............................................................................................................................................................................................................ucuuuccuaccccucccauC........................   2      1     LT-3
..............................................................................................................................................................................................................ucuuuccuaccccucccaua........................   42     0     LT-1
..............................................................................................................................................................................................................ucuuuccuaccccucccaua........................   41     0     LT-2
..............................................................................................................................................................................................................ucuuuccuaccccucccauU........................   2      1     LT-1
..............................................................................................................................................................................................................ucuuuccuaccccucccauac.......................   5      0     JCK-3
..............................................................................................................................................................................................................ucuuuccuaccccucccauac.......................   1      0     JCK-1
..............................................................................................................................................................................................................ucuuuccuaccccucccauac.......................   8      0     LT-2
..............................................................................................................................................................................................................ucuuuccuaccccucccauaA.......................   1      1     LT-1
..............................................................................................................................................................................................................ucuuuccuaccccucccauac.......................   4      0     LT-3
..............................................................................................................................................................................................................ucuuuccuaccccucccauac.......................   7      0     JCK-2
..............................................................................................................................................................................................................ucuuuccuaccccucccauac.......................   3      0     LT-1
..............................................................................................................................................................................................................uNuuuccuaccccucccauacc......................   1      1     JCK-3
..............................................................................................................................................................................................................ucuGuccuaccccucccauacc......................   1      1     JCK-1
..............................................................................................................................................................................................................uUuuuccuaccccucccauacc......................   1      1     JCK-3
..............................................................................................................................................................................................................ucuuuccuacUccucccauacc......................   87     1     LCK-1
..............................................................................................................................................................................................................ucuuuccuaccccucccauacc......................   226    0     LT-2
..............................................................................................................................................................................................................ucuuuccuaUcccucccauacc......................   3      1     LT-3
..............................................................................................................................................................................................................ucCuuccuaccccucccauacc......................   1      1     JCK-1
..............................................................................................................................................................................................................uNuuuccuaccccucccauacc......................   4      1     JCK-2
..............................................................................................................................................................................................................ucuuuccuaccccucccauacc......................   1146   0     JCK-1
..............................................................................................................................................................................................................ucuuuccuaccccucccauNcc......................   1      1     JCK-1
..............................................................................................................................................................................................................ucuuuccuaccccucccCuacc......................   6      1     JCK-2
..............................................................................................................................................................................................................ucuuuccuaccccucccauacc......................   1673   0     JCK-3
..............................................................................................................................................................................................................Ccuuuccuaccccucccauacc......................   1      1     JCK-3
..............................................................................................................................................................................................................ucuuuccuCccccucccauacc......................   2      1     LT-3
..............................................................................................................................................................................................................ucuuuccuaccccucccauacc......................   18     0     LCK-2
..............................................................................................................................................................................................................ucuuuccuaccccucccauCcc......................   3      1     JCK-2
..............................................................................................................................................................................................................ucuuuccuaccccucccauacU......................   1      1     LT-1
..............................................................................................................................................................................................................ucuuuccuaccccuUccauacc......................   1      1     JCK-3
..............................................................................................................................................................................................................ucuuuccuaccccucccauaGc......................   1      1     JCK-1
..............................................................................................................................................................................................................ucuuuccuaccccuccUauacc......................   2      1     LT-3
..............................................................................................................................................................................................................ucuuuccuaccccucccauacU......................   2      1     JCK-1
..............................................................................................................................................................................................................ucuuuccuacUccucccauacc......................   114    1     JT-1
..............................................................................................................................................................................................................ucuuCccuaccccucccauacc......................   1      1     JCK-3
..............................................................................................................................................................................................................ucuuuccuaccccucccGuacc......................   1      1     JCK-1
..............................................................................................................................................................................................................ucuuuccuacGccucccauacc......................   2      1     JCK-1
..............................................................................................................................................................................................................ucuuuccuacccAucccauacc......................   1      1     LCK-3
..............................................................................................................................................................................................................ucuuuccuCccccucccauacc......................   1      1     LT-1
..............................................................................................................................................................................................................ucuuuUcuaccccucccauacc......................   1      1     JCK-1
..............................................................................................................................................................................................................ucuuuccuaccccuUccauacc......................   4      1     JCK-1
..............................................................................................................................................................................................................ucuuuccuaAcccucccauacc......................   2      1     JCK-1
..............................................................................................................................................................................................................uNuuuccuaccccucccauacc......................   1      1     JCK-1
..............................................................................................................................................................................................................uUuuuccuaccccucccauacc......................   1      1     JCK-1
..............................................................................................................................................................................................................ucuuucUuaccccucccauacc......................   1      1     JCK-1
..............................................................................................................................................................................................................ucuuuccuaccccucUcauacc......................   1      1     LCK-3
..............................................................................................................................................................................................................ucuuuccuaccccuccUauacc......................   1      1     JCK-2
..............................................................................................................................................................................................................ucuuuUcuaccccucccauacc......................   4      1     JCK-2
..............................................................................................................................................................................................................ucuuuccuaccccucccauaGc......................   1      1     JCK-2
..............................................................................................................................................................................................................ucuuuccuacUccucccauacc......................   80     1     LCK-2
..............................................................................................................................................................................................................ucCuuccuaccccucccauacc......................   1      1     LT-2
..............................................................................................................................................................................................................ucuuuccuaccccucccauacc......................   434    0     LT-1
..............................................................................................................................................................................................................ucuuuccuaccccucccauacc......................   29     0     JT-2
..............................................................................................................................................................................................................ucuuuccuaccccucccCuacc......................   2      1     JCK-3
..............................................................................................................................................................................................................ucuuuccuaccNcucccauacc......................   3      1     JCK-1
..............................................................................................................................................................................................................ucuuuccuaccccucccCuacc......................   4      1     JCK-1
```

```
                                                                                                                                Star                                                                         Mature
ccuuuuuuucaccaaaaucuagacucaauuaauuucucuuuucccuuccuccaccaucugguguauuucuuuucucacuaaaaggauggaagagggaaaggcuuaagaguguggguggaagugauagguauggggguuaggacagagagaaauacucucuuuuucccaauuuauuuauuuuuuaauuggggggaagggaaugaauucuuuccuaccccucccauaccauucauuuccauccuuuccuuc
.............................................................................................................................................................................................................ucuuuccuacUccucccauacc......................  26  1  JT-3
.............................................................................................................................................................................................................ucuuuccuaccccucccCuacc......................  1  1  LT-2
.............................................................................................................................................................................................................ucuuuccuaccccucccauacc......................  214  0  LCK-3
.............................................................................................................................................................................................................ucuuuccuaccccucccauCcc......................  1  1  JCK-1
.............................................................................................................................................................................................................ucuuuccuaccccucccauCcc......................  1  1  LT-1
.............................................................................................................................................................................................................ucuuuccuaccccucccauCcc......................  2  1  LT-3
.............................................................................................................................................................................................................ucuuuccuacUccucccauacc......................  14  1  JT-2
.............................................................................................................................................................................................................ucuuuccuaccccucccauaUc......................  1  1  JCK-2
.............................................................................................................................................................................................................ucuuuccuaccccucccauacc......................  26  0  LCK-1
.............................................................................................................................................................................................................Ccuuuccuaccccucccauacc......................  3  1  JCK-2
.............................................................................................................................................................................................................Ccuuuccuaccccucccauacc......................  2  1  LT-2
.............................................................................................................................................................................................................ucuuuccuaccccucccauacc......................  419  0  LT-3
.............................................................................................................................................................................................................ucuuucUuaccccucccauacc......................  3  1  JCK-2
.............................................................................................................................................................................................................ucuuuccuacGccucccauacc......................  3  1  JCK-3
.............................................................................................................................................................................................................ucuCuccuaccccucccauacc......................  1  1  LT-3
.............................................................................................................................................................................................................ucuuuccuacUccucccauacc......................  1  1  JCK-2
.............................................................................................................................................................................................................ucuuuccuacGccucccauacc......................  2  1  JCK-2
.............................................................................................................................................................................................................ucuuuccuaccccCcccauacc......................  1  1  JCK-2
.............................................................................................................................................................................................................ucuuuccuCccccucccauacc......................  3  1  JCK-3
.............................................................................................................................................................................................................ucuuuccuaccccucccauaUc......................  1  1  JCK-3
.............................................................................................................................................................................................................ucuuuccuaccccucccauacA......................  1  1  JCK-2
.............................................................................................................................................................................................................ucuuuccuaccccucccauacc......................  48  0  JT-1
.............................................................................................................................................................................................................ucuuuccuCccccucccauacc......................  7  1  JCK-2
.............................................................................................................................................................................................................ucCuuccuaccccucccauacc......................  1  1  JCK-2
.............................................................................................................................................................................................................ucuuuccuaccccucccauCcc......................  1  1  LT-2
.............................................................................................................................................................................................................ucuuuccuaccccucccauacc......................  2190  0  JCK-2
.............................................................................................................................................................................................................ucCuuccuaccccucccauacc......................  1  1  JCK-3
.............................................................................................................................................................................................................ucuuuccuaccccucccauacc......................  18  0  JT-3
.............................................................................................................................................................................................................uUuuuccuaccccucccauacc......................  3  1  JCK-2
.............................................................................................................................................................................................................ucuuuccuaccccucccauacca.....................  1  0  JCK-1
.............................................................................................................................................................................................................ucuuuccuaccccucccauaccU.....................  11  1  LT-1
.............................................................................................................................................................................................................ucuuuccuaccccucccauacca.....................  1  0  LT-1
.............................................................................................................................................................................................................ucuuuccuaccccucccauaccU.....................  6  1  JCK-1
.............................................................................................................................................................................................................ucuuuccuaccccucccauaccU.....................  2  1  JCK-3
.............................................................................................................................................................................................................ucuuuccuaccccucccauaccU.....................  5  1  LT-3
.............................................................................................................................................................................................................ucuuuccuaccccucccauacca.....................  2  0  LT-3
.............................................................................................................................................................................................................ucuuuccuaccccucccauacca.....................  1  0  JCK-2
.............................................................................................................................................................................................................ucuuuccuaccccucccauaccU.....................  2  1  JCK-2
.............................................................................................................................................................................................................ucuuuccuaccccucccauaccaA....................  3  1  LT-2
.............................................................................................................................................................................................................ucuuuccuaccccucccauaccUu....................  6  1  LCK-3
.............................................................................................................................................................................................................ucuuuccuaccccucccauaccUu....................  2  1  LT-2
.............................................................................................................................................................................................................ucuuuccuaccccucccauaccUuu...................  3  1  LT-2
.............................................................................................................................................................................................................ucuuuccuaccccucccauaccUuu...................  1  1  LT-3
.............................................................................................................................................................................................................ucuuuccuaccccucccauaccauuG..................  1  1  LT-1
.............................................................................................................................................................................................................ucuuuccuaccccucccauaccaCuc..................  1  1  JCK-2
..............................................................................................................................................................................................................cuuuccuaccccucccau.........................  2  0  LCK-3
..............................................................................................................................................................................................................cuuuccuaccccucccau.........................  1  0  JCK-3
..............................................................................................................................................................................................................cuuuccuaccccucccaua........................  1  0  JCK-3
..............................................................................................................................................................................................................cuuuccuaccccucccaua........................  1  0  JCK-1
..............................................................................................................................................................................................................cuuuccuaccccucccaua........................  4  0  LCK-3
..............................................................................................................................................................................................................cuuuccuaccccucccauacc......................  6  0  LT-3
..............................................................................................................................................................................................................cuuuccuaccccucccauacc......................  11  0  JCK-2
..............................................................................................................................................................................................................cuuuccuaccccucccauacc......................  11  0  JCK-1
..............................................................................................................................................................................................................cuuuccuaccccucccauacc......................  16  0  JCK-3
..............................................................................................................................................................................................................cuuuccuaccccucccauaccauuU..................  2  1  JCK-2
..............................................................................................................................................................................................................cuuuccuaccccucccauaccauuU..................  2  1  JCK-3
...............................................................................................................................................................................................................uuuccuaccccucccauU........................  1  1  LT-3
...............................................................................................................................................................................................................uuuccuaccccucccaua........................  2  0  LT-3
...............................................................................................................................................................................................................uuuccuaccccucccaua........................  4  0  LCK-3
...............................................................................................................................................................................................................uuuccuaccccucccaua........................  1  0  LT-1
...............................................................................................................................................................................................................uuuccuaccccucccaua........................  1  0  LT-2
...............................................................................................................................................................................................................uuuccuaccccucccaua........................  5  0  JCK-3
...............................................................................................................................................................................................................uuuccuaccccucccaua........................  1  0  JCK-2
...............................................................................................................................................................................................................uuuccuaccccucccauacc......................  8  0  LT-2
...............................................................................................................................................................................................................uuuccuaccccucccauacU......................  1  1  LT-1
...............................................................................................................................................................................................................uuuccuaccccucccauacc......................  11  0  LCK-3
...............................................................................................................................................................................................................Cuuccuaccccucccauacc......................  2  1  LT-2
...............................................................................................................................................................................................................uuuccuaccccucccauacc......................  10  0  JCK-3
...............................................................................................................................................................................................................uuuccuaccccucccauacc......................  28  0  LT-3
...............................................................................................................................................................................................................uuuccuaccccucccauacc......................  33  0  LT-1
```

Star Mature

```
ccuuuuuuucaccaaaaucuagacucaauuaauuucucuuuucccuuccuccaccaucugguguauuucuuuucucacuaaaaggauggaagagggaaaggcuuaagaguguggguggaagugauagguaugggggguuaggacagagagaaauacucucuuuuucccaauuuauuuauuuuuuaauuggggggaagggaaugaauucuuuccuaccccucccauaccauucauuuccauccuuuccuuc
................................................................................................................................................................................................................uuuccuaccccucccauacc......................   12   0   JCK-1
................................................................................................................................................................................................................uuuccuaccccucccauacc......................   18   0   JCK-2
................................................................................................................................................................................................................uuuccuCccccucccauacc......................   2   1   LT-3
................................................................................................................................................................................................................uuuccuaccccucccauacca.....................   2   0   JT-1
................................................................................................................................................................................................................uuuccuaccccucccauacca.....................   3   0   LCK-3
................................................................................................................................................................................................................uuuccuaccccucccauacca.....................   1   0   JCK-1
................................................................................................................................................................................................................uuuccuaccccucccauaccU.....................   1   1   JCK-1
................................................................................................................................................................................................................uuuccuaccccucccauacca.....................   2   0   LT-1
................................................................................................................................................................................................................uuuccuaccccucccauaccU.....................   1   1   JCK-3
................................................................................................................................................................................................................uuuccuaccccucccauacca.....................   1   0   LT-3
................................................................................................................................................................................................................uuuccuaccccucccauaccU.....................   2   1   LT-1
................................................................................................................................................................................................................uuuccuaccccucccauacca.....................   1   0   JCK-2
................................................................................................................................................................................................................uuuccuaccccucccauaccU.....................   1   1   JCK-2
................................................................................................................................................................................................................uuuccuaccccucccauacca.....................   2   0   JCK-3
................................................................................................................................................................................................................uuuccuaccccucccauaccau....................   6   0   JT-3
................................................................................................................................................................................................................uuuccuaccccucccauaccUu....................   1   1   LT-1
................................................................................................................................................................................................................uuuccuaccccucccauaccau....................   7   0   JCK-3
................................................................................................................................................................................................................uuuccuacccAucccauaccau....................   1   1   JCK-2
................................................................................................................................................................................................................uuuccuaccccucccauaccau....................   4   0   LT-1
................................................................................................................................................................................................................uuuccuaccccucccauaccau....................   10   0   JCK-1
................................................................................................................................................................................................................uuuccuaccccucccauaccau....................   5   0   JT-2
................................................................................................................................................................................................................uNuccuaccccucccauaccau....................   1   1   JCK-2
................................................................................................................................................................................................................uuuccuaccccucccauaccau....................   15   0   JCK-2
................................................................................................................................................................................................................uuuccuaccccucccauaccau....................   2   0   LCK-3
................................................................................................................................................................................................................uuuccuaccccucccauaccau....................   7   0   LT-3
................................................................................................................................................................................................................uuuccuaccccucccauaccauu...................   3   0   JCK-2
................................................................................................................................................................................................................uuuccuaccccucccauaccUuu...................   2   1   LT-2
................................................................................................................................................................................................................uuuccuacccGucccauaccauu...................   1   1   LT-2
................................................................................................................................................................................................................uuuccuaccccucccauaccauu...................   3   0   LT-1
................................................................................................................................................................................................................uuuccuaccccucccauaccauu...................   2   0   LT-2
................................................................................................................................................................................................................uuuccuaccccNcccauaccauu...................   1   1   JCK-1
................................................................................................................................................................................................................uuuccuaccccucccauaccauu...................   3   0   LT-3
................................................................................................................................................................................................................uuuccuaccccucccauaccauu...................   2   0   JCK-3
................................................................................................................................................................................................................uuuccuaccccucccauaccauuc..................   1   0   LCK-3
................................................................................................................................................................................................................uuuccuaccccucccauaccauuc..................   3   0   JCK-2
................................................................................................................................................................................................................uuuccuaccccucccauaccauuU..................   1   1   LT-1
................................................................................................................................................................................................................uuuccuaccccucccauaccauuc..................   6   0   JCK-3
................................................................................................................................................................................................................uuuccuaccccucccauaccauuc..................   1   0   LT-3
................................................................................................................................................................................................................uuuccuaccccucccauaccauuc..................   3   0   LT-1
................................................................................................................................................................................................................uuuccuaccccucccauaccauuc..................   1   0   JCK-1
.................................................................................................................................................................................................................uuccuaccccucccauacc......................   6   0   LCK-3
.................................................................................................................................................................................................................uuccuaccccucccauacc......................   1   0   LT-3
.................................................................................................................................................................................................................uuccuaccccucccauacc......................   8   0   JT-1
.................................................................................................................................................................................................................uuccuaccccucccauacc......................   3   0   LT-1
.................................................................................................................................................................................................................uAccuaccccucccauaccauu...................   2   1   JCK-3
.................................................................................................................................................................................................................uuccuaccccucccauaccauu...................   2   0   LT-1
.................................................................................................................................................................................................................uuccuaccccucccauaccauu...................   5   0   LCK-3
.................................................................................................................................................................................................................uAccuaccccucccauaccauu...................   2   1   JCK-1
.................................................................................................................................................................................................................uuccuaccccucccauaccauu...................   1   0   JCK-2
.................................................................................................................................................................................................................uuccuaccccucccauaccauu...................   2   0   JCK-3
.................................................................................................................................................................................................................uuccuaccccucccauaccauuc..................   11   0   JCK-2
.................................................................................................................................................................................................................uuccuaccccucccauaUcauuc..................   1   1   JCK-2
.................................................................................................................................................................................................................uuccuaccccucccauaccauuc..................   4   0   JCK-1
.................................................................................................................................................................................................................uuccuaccccucccauaccauuc..................   4   0   JCK-3
.................................................................................................................................................................................................................uuccuaccccucccauaccauuU..................   1   1   LT-1
..................................................................................................................................................................................................................uccuaccccucccauacc......................   1   0   LT-1
..................................................................................................................................................................................................................uccuaccccucccauacc......................   2   0   LCK-3
..................................................................................................................................................................................................................uccuaccccucccauacc......................   1   0   LT-2
..................................................................................................................................................................................................................uccuaccccucccauacc......................   1   0   JCK-2
..................................................................................................................................................................................................................uccuaccccucccauacc......................   2   0   LT-3
..................................................................................................................................................................................................................uccuaccccucccauacc......................   1   0   JCK-1
..................................................................................................................................................................................................................uccuaccccucccauacc......................   5   0   JT-3
..................................................................................................................................................................................................................uccuaccccucccauacc......................   3   0   JT-2
..................................................................................................................................................................................................................uccuaccccucccauacca.....................   5   0   LT-2
..................................................................................................................................................................................................................uccuaccccucccauacca.....................   5   0   JT-3
..................................................................................................................................................................................................................uccuaccccucccauaccauu...................   52   0   JCK-1
..................................................................................................................................................................................................................uccuaccccucccauaccauu...................   11   0   LT-3
..................................................................................................................................................................................................................uccuaccccucccauaccauu...................   20   0   LT-1
..................................................................................................................................................................................................................uccuaccccucccauaccauu...................   5   0   LT-2
..................................................................................................................................................................................................................uccuacccAucccauaccauu...................   1   1   JCK-2
```

Star (uauggggguuaggacagaga) — **Mature** (ucuuuccuaccccucccauacc)

```
ccuuuuuuucaccaaaaucuagacucaauuaauuucucuuuucccuuccuccaccaucugguguauuucuuuucucacuaaaaggauggaagagggaaaggcuuaagagugugggugga agugauagguauggggguuaggacagagagaaauacucucuuuucccaauuuauuuauuuuuuaauugggggaagggaaugaauucuuuccuaccccucccauaccauucauuuccauccuuuccuuc
```

uccuaccccucccauaccauu	85	0	JCK-2
uccuaccccucccauaccCuu	1	1	JCK-1
uccuaccccucccauaccauu	53	0	JCK-3
uNcuaccccucccauaccauu	1	1	JCK-3
uccuaccccucccaCaccauu	1	1	JCK-2
uccuaccccucccauaccauuA	1	1	JCK-3
uccuaccccucccauaccauuU	5	1	JT-3
uccuaccccucccauaccauuc	8	0	LCK-3
uccuaccccucccauCccauuc	1	1	JCK-1
uccuaccccucccauaccauuU	1	1	LT-1
uccuaccccucccauaccauuU	2	1	LT-3
uccuaccccucccauaccauuc	101	0	JCK-3
uccuaccccucccauaccauuU	3	1	JCK-1
uccuaccccucccauaccauuc	16	0	LT-1
uccuaccccucccauaccauuU	6	1	JCK-2
uccuaccccucccauaccauuU	3	1	JT-2
Cccuaccccucccauaccauuc	1	1	JCK-2
uccuaccccucccCuaccauuc	1	1	JCK-2
uccuacccAucccauaccauuc	1	1	JCK-3
uccuaccccucccauaccauuU	3	1	JCK-3
uccuaccccucccauaccauuc	66	0	JCK-1
uccuaccccucccauaccauuc	23	0	LT-3
uccuCcccucccauaccauuc	1	1	JCK-2
uccuaccccucccauaccauuc	142	0	JCK-2
uccuaccccucccauaccauuc	5	0	LT-2
uccuaccccuAccauaccauuc	1	1	JCK-1
ccuaccccucccauacca	2	0	LT-2
ccuaccccucccauacca	1	0	LT-3
ccuaccccucccauaccau	3	0	JCK-3
ccuaccccucccauaccauu	12	0	JCK-3
ccuaccccucccauaccauu	5	0	JCK-1
ccuaccccucccauaccauu	1	0	LCK-3
ccuaccccucccauaccauu	3	0	LT-1
cUuaccccucccauaccauu	1	1	JCK-2
ccuaccccucccauaccauu	3	0	LT-3
ccuaccccucccauaccauu	20	0	JCK-2
ccuaccccucccauaccauu	4	0	LT-2
ccuaccccucccauaccauuc	5	0	LT-3
ccuaAcccucccauaccauuc	1	1	JCK-1
ccuaccccCcccauaccauuc	1	1	JCK-2
ccuaccccucccauaccauuc	34	0	JCK-2
ccuaccccucccauaccauuU	2	1	JCK-1
ccuaccccucccauaccauuU	1	1	LT-3
ccuaccccucccauaccauuU	5	1	JCK-2
ccuaccccucccauaccauuc	4	0	LT-1
ccuaccccucccauaccauuc	22	0	JCK-1
ccuaccccucccauaccauuc	32	0	JCK-3
ccuaccccucccauaccauuU	5	1	JCK-3
ccuaccccucccauaccauucU	3	1	LT-2
ccuaccccucccauaccauuca	1	0	JCK-1
cuaccccucccauaccau	3	0	LT-1
cuaccccucccauaccauu	1	0	LT-2
cuaccccucccauaccauu	5	0	LT-3
Uuaccccucccauaccauu	1	1	LT-1
cuaccccucccauaccaCu	1	1	LT-2
cuaccccucccauaccauu	4	0	LCK-3
cuaccccucccauaccauu	6	0	LT-1
cuaccccucccauaccauu	3	0	JCK-2
cuaccccucccauaccauuU	1	1	LT-2
cuaccccucccauaccauuU	1	1	LT-1
cuaccccucccauaccauuc	4	0	LT-1
cuaccccucccauaccauuU	2	1	JCK-3
cuaccccucccauaccauuc	6	0	LT-3
cuaccccucccauaccauuc	5	0	LT-2
cuaNcccucccauaccauuc	1	1	JCK-1
cuaccccucccauaccauuU	1	1	JCK-2
cuaccccucccauaccauuc	3	0	JCK-3
cuaccccucccauaccauuc	5	0	JCK-2
cuCcccucccauaccauuc	1	1	LT-3
cuaccccucccauaccauuU	3	1	LCK-3

Star Mature

ccuuuuuuucaccaaaaucuagacucaauuaauuucucuuuucccuuccuccaccaucugguguauuucuuuucucacuaaaaggauggaagagggaaaggcuuaagagugugggugggaagugauagguauggggguuaggacagagagaaauacucucuuuuucccaauuuauuuauuuuuuaauugggggaagggaaugaauucuuuccuaccccucccauaccauucauuuccauccuuuccuuc

cuaccccucccauaccauuc	3	0	JCK-1
cuaccccucccauaccauuca	1	0	LT-1
cuaccccucccauaccauucU	1	1	LCK-3
cuaccccucccauaccauuca	2	0	JCK-1
uaccccucccauaccauuc	1	0	LT-1
ccauaccauucauuuccauccu	2	0	LT-1
ccauaccauucauuuccauccuAu	1	1	JCK-2
ccauaccauucauuuccauccuAu	1	1	JCK-3
cauaccauucauuuccauU	1	1	JCK-2
cauaccauucauuuccauc	1	0	LT-1
cauaccauucauuuccauccu	1	0	LT-3
cauaccauucauuuccauccuu	1	0	JCK-2
cauaccauucauuuccauccuu	2	0	JCK-3
cauaccauucauuuccauccuuu	1	0	JCK-2
cauaccauucauuuccauccuuu	2	0	JCK-3
cauaccauucauuuccauccuuuc	2	0	JCK-1
auaccauucauuuccauc	1	0	LT-3
auaccauucauuuccauccuu	1	0	LT-3
auaccauucauuuccauccuuu	1	0	LT-3
auaccauucauuuccauccuuuU	1	1	JCK-3
auaccauucauuuccauccuuuc	1	0	LT-2
auaccauucauuuccauccuuuc	1	0	LT-1
auaccauucauuuccauccuuucc	3	0	JCK-2
auaccauucauuuccCuccuuucc	1	1	JCK-2
auaccauucauuuccauccuuuccuu	1	0	LT-3
uaccauucauuuccauccuuucc	2	0	JCK-1
auucauuuccauccuuucc	2	0	LT-3
auucauuuccauccuuuccu	2	0	LT-2
auucauuuccauccuuuccuu	3	0	LT-3
